# Nutrients, Genetic Factors, and Their Interaction in Non-Alcoholic Fatty Liver Disease and Cardiovascular Disease

**DOI:** 10.3390/ijms21228761

**Published:** 2020-11-19

**Authors:** Rosa Lombardi, Federica Iuculano, Giada Pallini, Silvia Fargion, Anna Ludovica Fracanzani

**Affiliations:** 1 Department of Pathophysiology and Transplantation, University of Milan, 20122 Milan, Italy; rosa.lombardi@unimi.it (R.L.); federica.iuculano@unimi.it (F.I.); giada.pallini@unimi.it (G.P.); silvia.fargion@unimi.it (S.F.); 2 Ca’ Granda IRCCS Foundation, Policlinico Hospital of Milan, 20122 Milan, Italy

**Keywords:** nutrigenomic, fructose, PUFA, micronutrients, cardiovascular disease, NAFLD, diet, epigenetic, PNPLA3, genetic influence

## Abstract

Non-alcoholic fatty liver disease (NAFLD) is the most common chronic liver disease in Western countries and expose patients to increased risk of hepatic and cardiovascular (CV) morbidity and mortality. Both environmental factors and genetic predisposition contribute to the risk. An inappropriate diet, rich in refined carbohydrates, especially fructose, and saturated fats, and poor in fibers, polyunsaturated fats, and vitamins is one of the main key factors, as well as the polymorphism of patatin-like phospholipase domain containing 3 (PNPLA3 gene) for NAFLD and the apolipoproteins and the peroxisome proliferator-activated receptor (PPAR) family for the cardiovascular damage. Beyond genetic influence, also epigenetics modifications are responsible for various clinical manifestations of both hepatic and CV disease. Interestingly, data are accumulating on the interplay between diet and genetic and epigenetic modifications, modulating pathogenetic pathways in NAFLD and CV disease. We report the main evidence from literature on the influence of both macro and micronutrients in NAFLD and CV damage and the role of genetics either alone or combined with diet in increasing the risk of developing both diseases. Understanding the interaction between metabolic alterations, genetics and diet are essential to treat the diseases and tailoring nutritional therapy to control NAFLD and CV risk.

## 1. Introduction

Non-alcoholic fatty liver disease is defined by the presence of fat in more than 5% of hepatocytes without excessive alcohol consumption and after exclusion of all other causes of liver disease. It is the most common chronic liver disease in Western countries [1] and includes a group of hepatic disorders ranging from simple steatosis to more progressive forms such as non-alcoholic steatohepatitis (NASH), characterized by hepatic inflammation, and hepatic fibrosis, possibly leading to NAFLD-related cirrhosis and hepatocellular carcinoma.

NAFLD is characterized by metabolic alterations, mostly overweight and central obesity, dyslipidemia, hypertension, insulin-resistance, and type 2 diabetes mellitus so that the acronym NAFLD has been substituted with the term MAFLD (i.e., metabolic associated fatty liver disease) [2]. However, given the really recent coin of MAFLD term and the almost totality of literature data on fatty liver disease referred as NAFLD, in the present review, we’ll keep using the acronym NAFLD. 

This metabolic imbalance exposes NAFLD patients to increased cardiovascular (CV) risk, so that the leading cause of mortality in patients with NAFLD is cardiovascular disease (CVD) [3]. Besides coronary artery disease, patients with NAFLD present also higher rate of atrial fibrillation, heart failure, atherosclerosis, altered arterial stiffness, and myocardial remodeling [4]. 

Actually, NAFLD and CVD share common pathogenetic risk factors. Insulin resistance is one of the key factors in the development of both diseases, prompting increased circulation of free fatty acids (FFAs) to the liver and imbalance in lipid metabolism, with consequent hepatic and vessels fat accumulation [5]. Increased lipid accumulation fosters mitochondrial dysfunction by lipo-toxicity and activation of oxidative stress, both in the liver and in peripheral arteries. Moreover, impairment in lipid metabolism enhances hepatic production of adipokines and inflammatory cytokines, as well as of procoagulant factors. In addition, diet may alter gut microbiota, which is involved in bile acid metabolism and systemic and hepatic inflammation [1].

Both environmental factors and genetic predisposition play a central role in sustaining the above-mentioned pathogenetic mechanisms. Among environmental factors, an inappropriate diet, rich in refined carbohydrates, especially fructose, and saturated fats, and physical inactivity are the key factors. As for the genetic substratum, the polymorphism of I148M PNPLA3 gene (patatin-like phospholipase domain containing 3) is the major determinant of NAFLD, whilst the transmembrane 6 superfamily member 2 gene (TM6SF2), the membrane bound O-acyltransferase domain-containing 7 gene (MBOAT7), and the glucokinase regulatory protein (GCKR) variants play a minor role [6]. Conversely, TM6SF2 mutation seems to reduce CV risk [7]. Along with genetic alterations, epigenetics modifications are also responsible for various clinical manifestations of both hepatic and CVD.

Nowadays, no established pharmacological treatment is available for NAFLD, so that modification in lifestyle, especially diet and regular physical exercise, are standard of care. Similarly, the same approach is advisable to prevent and manage CVD. Many dietary approaches have been proposed and the Mediterranean diet has recently been shown to have a role in improving risk factors associated with metabolic syndrome and NAFLD [8], as well as in reducing CV events [9]. 

Given the complexity of NAFLD and cardiovascular disease, understanding the interaction between metabolic alterations, genetics, and diet, is essential to treat the diseases and tailoring nutritional therapy to control NAFLD and CV risk.

The aim of our review is to explore the impact of macro and micro-nutrients on development and progression of NAFLD and to assess their influence on CV risk, as well as the interplay between micro/macro-nutrients and genetic substrate in the etiopathogenetic pathway. Finally, we will also report our preliminary data of an observational study conducted at the Outpatient Hepatology Service of Policlinico Hospital of Milan evaluating the impact of macro and micronutrients on liver disease and cardiovascular damage in patients with NAFLD.

## 2. Role of Carbohydrates in NAFLD and Cardiovascular Disease

### 2.1. Carbohydrates and NAFLD

The Mediterranean diet is one of the mostly suggested dietetic regimen for patients with NAFLD and is characterized by reduced carbohydrate intake (40% of the calories vs. 50–60% in a typical low-fat diet), with particular decrease of sugars and refined carbohydrates [9]. 

Carbohydrates are classified into monosaccharides (fructose, glucose, and galactose) and disaccharides. Fructose is naturally present in fruits; however, it is often present in the form of added sugar, as a sweetener to other products such as soft drinks, biscuits, and bakery products in the form of sucrose (made of 50% of glucose and 50% of fructose) and high fructose corn syrup (HFCS) (with up to 55% of fructose). 

Fructose has been demonstrated as the main carbohydrate determinant in hepatic fat accumulation. In fact, Schwarz et al. [10] demonstrated a higher fat accumulation in patients exposed to a high-fructose diet compared to those with a diet poor in fructose and rich in complex carbohydrates like cereals, bread, pasta, rice, and potatoes.

Indeed, fructose exerts a negative metabolic effect promoting de novo lipogenesis (DNL) and hepatic fat accumulation [11] either directly, being a substrate of DNL, or leading to a hyperproduction of Acetyl-Co-A from citrate, another substrate for DNL as well as a powerful activator of key enzymes in the DNL itself [12]. In addition, the higher intake of fructose fosters oxidative stress and hepatic insulin resistance [13], which, along with intrahepatic fat accumulation, promote the development of hepatic steatosis and cardiometabolic events [14,15]. Moreover, fructose inhibits the production of insulin and leptin, hormones which modulate the feeling of satiety, fostering increased energy intake and weight gain. Finally, fructose ingestion alters gut microbiota, with a reduced capacity to degrade carbohydrates to short-chain fatty acids, and increases gut permeability promoting metabolic dysfunction [16].

Population studies revealed a close association between overconsumption of added sugar (e.g., sucrose and high-fructose corn syrup) and the presence of NAFLD in both adults and children [17,18,19]. In particular, the Framingham Heart Study, involving more than 2600 subjects examined by abdomen computer tomography for the presence of NAFLD, showed a dose–response association between soft drinks consumption and fatty liver disease [20]. In addition, soft-drinks’ usual consumers with NAFLD have a higher rate of inflammation and fibrosis [21] compared to non-consumers, and an improvement of liver histology consequent to fructose restriction has been demonstrated [22]. In contrast, Cortez-Pinto et al. [23] reported in a cohort of 45 biopsy proven NAFLD patients a higher consumption of fat and less carbohydrate in NASH subjects compared to non-NASH ones, with only a slight increase in the consumption of fructose in those with more severe histological features. 

Besides observational data, a large number of interventional studies explored the role of carbohydrates in patients with NAFLD. 

Solid data demonstrated that consumption of a high-fructose diet even for a short term (i.e., 1–2 weeks) significantly favor hepatic fat deposition [24,25,26], possibly because of the stimulation of DNL by fructose, especially when combined with glucose ingestion. The role of fructose in DNL has been confirmed by a Sevastianova et al. [27] who demonstrated that a 3-week hypercaloric (+1000 kcal/day) diet enriched with sugars (i.e., candy, fruit juice, and sugar-sweetened beverages) led to an increase in fatty liver and was associated with an increase in the secretion of very low density lipoprotein and an increase in the lipogenic index, (i.e., the ratio of palmitate to linoleate acid), an indirect measure of DNL. 

Another study demonstrated the specific steatogenic role of fructose showing that a highly hypercaloric diet, containing a low amount of fructose, did not elicit an increase in liver fat content [24].

Consistently with these results, Maersk et al. [14] compared the effects of ingesting 1 L/day of a sugar-sweetened beverage with a calorie-matched milk for six months and showed that sugar-sweetened beverage significantly increased liver fat by approximately 1.5 fold, despite similar calorie intake and weight gain. 

While the causative role of fructose in hypercaloric diet has a consistent evidence, data on the impact of carbohydrates on liver fat accumulation in the setting of hypo or isocaloric diet are scarce and with contrasting results. One study involving NAFLD patients exposed to hypocaloric diet reported a more evident reduction in liver fat content obtained by reducing carbohydrate intake compared to the reduction of total calories without changing the macronutrient composition of the diet [28]. In contrast, Haufe et al. and Volynets et al. [29,30] suggested that total calorie deficit is the mediating factor for decreasing liver fat and that the carbohydrate intake has little influence. Likewise, also data from isocaloric diets enriched with added sugars did not show an influence on liver fat content which vice-versa was highlighted in subjects assuming hypercaloric high-fructose diets, possibly because of increased substrate availability for DNL.

Along with its specific effect, fructose pathogenetic action is modulated by co-ingestion of other macronutrients. Sobrecases et al. showed in a study comparing a 7-day hypercaloric diet enriched with fructose or saturated fatty acids (SFA) that the latter increased liver fat to a greater extent than fructose (~86% vs. ~16% change in hepatic fat content), and that SFA also had an additive effect on hepatic fat accumulation exerted by sugars [26]. On the other hand, supplementation of high-fructose diet with small amount of proteins (6.6 g of essential amino acids/day) leads to an attenuation of hepatic fat accumulation [31]. The beneficial effect of proteins may rely on the augmented fatty acid oxidation due to the increased energy required by the metabolism of amino acids (i.e., gluconeogenesis and cycle of urea) [32]. 

Finally, the timing of food intake also affects hepatic fat deposition, being more dangerous when consumed between rather than alongside meals [33]. 

### 2.2. Carbohydrates and Cardiovascular Disease

As for the impact in the development of NAFLD, fructose has been demonstrated to exert a negative effect on CV risk. Interestingly, the amount of added sugar, consumed mainly as HFCS, dramatically increased during the last few decades in Europe and the USA [34] paralleling the augmented prevalence of coronary artery disease (CAD) [35], as supported by a prospective study involving a large female cohort [36]. The association between HCFC and CVD has been confirmed by consistent data. A prospective study involving a cohort of adults in the USA showed that added sugar intake was a risk factor for CV mortality even when adjusted for conventional cardiovascular risk factors such hypertension and dyslipidemia [37]. Another prospective study involving more than 2600 Iranian subjects, without a history of CV events followed-up for approximately seven years, demonstrated that consumption of food with added fructose, but not natural sugars, was associated with an increased risk of CV events, either myocardial infarction or stroke, and cardiovascular mortality. This result points out that only industrial sweetened products expose to cardiovascular risk, whilst consumption of natural fructose food, like fresh fruit, is safe. In another population-based cohort study of nearly 40,000 adults, daily soft drink consumption increased the risk of CV events by 21% [38] and in the UK, The National Health Service (NHS) showed that sugar-sweetened beverages significantly increased the risk of stroke by 16% per one serving per day and coronary heart disease by 20% [39].

As a consequence, European guidelines for the management of CVD strongly discourage the intake of added sugars in diet, especially sweetened beverages, allowing no more than 25 g per day [40]. 

One underlying mechanism which may explain the adverse cardiometabolic outcome of fructose intake is the induction of hepatic lipogenesis and increased circulatory triglycerides levels, as well as the promotion of insulin resistance and oxidative stress [41], as already mentioned in the previous paragraph.

As a confirmation of the deleterious effect of added sugars on cardiovascular risk, in a 4-week prospective randomized study, 24 Hispanic overweight adolescents with hepatic steatosis, all usual high fructose containing beverages drinkers, were randomized to fructose-drinks vs. glucose only beverages. After the intervention period, patients randomized to glucose beverage presented a significant improvement in the cardiometabolic profile (plasma C-reactive protein, insulin-resistance indexes, plasma free fatty acids, and circulating oxydated low-density lipoprotein levels) compared to baseline and to those in the fructose-beverage arm of the study [42]. 

Conversely to fructose, whole grains seem to have a protective role on cardiovascular risk, mainly due to the high concentration of fibers. Fibers reduce inflammation and reactive oxidation and improve lipid profiles and blood pressure [43]. Ultimately, they inhibit absorption of fats and simple carbohydrates, with improvement of the cardiometabolic profile. 

A small randomized clinical trial including a cohort of 300 participants explored the impact of a diet enriched with whole grain on surrogate markers of cardiovascular disease such as blood pressure, cholesterol, and markers of inflammation, and demonstrated a significant improvement of all of them [44]. In addition, a meta-analysis of seven prospective studies showed that an increased consumption of whole grain prompted a 21% reduction in cardiovascular disease events and mortality [45]. 

## 3. Role of Lipids in NAFLD and Cardiovascular Disease

### 3.1. Lipids and NAFLD

Fatty acids are long hydrocarbon chains and are divided in four categories: saturated fatty acids (SFAs), without double bound between carbon atoms, monounsaturated fatty acids (MUFAs), with only one double bound, polyunsaturated fatty acids (PUFAs), with more than one double bound, and trans fatty acids (TFAs), when the two hydrogen atoms are on opposite sides of the double bond [46]. Among PUFAs, n-3 PUFAs and n-6 PUFAs are essential because humans cannot synthesize them and their precursors (linoleic (18:2 n-6) acid and linolenic acid (18:3 n-3) need to be introduced in the diet, to build longer carbon chains [46]. Indeed, if on one hand a great supply of linolenic acid is beneficial, on the other hand, evidence is rising on the need of reducing that of linoleic acid which may favor inflammation and metabolic diseases [47]. 

In the last century, fatty acids consumption in the daily life has dramatically changed from a diet rich in MUFAs, principally found in olive oil, and, in PUFAs, contained in fish and shellfish, vegetable oils, seeds, and nuts, to a Westernized dietary, where SFAs and TFAs, present in meat and dairy products, prevail [48].

Regarding the consumption of fatty acids, Donnelly et al. demonstrated that 15% of hepatic fat derived from diet and that this percentage could increase if dietary fat consumption was higher than 30% of daily energy requirements [49]. Moreover, two epidemiological studies by Toshimitsu at al. and Musso at al. illustrated that NASH patients had a diet richer in saturated fat and cholesterol and poorer in PUFAs compared to the diet of age, gender, and BMI matched controls without NASH [50,51]. Analyzing PUFA intake, a lower consumption of n-3 PUFA and a higher n-6/n-3 ratio were observed in NAFLD and NASH patients compared to controls [23]. Moreover, patients with NAFLD had a depletion in blood and tissue PUFAs content, especially those of marine origin, the n-3 PUFAs (eicosapentaenoic acid (EPA) and docosahexaenoic acid (DHA)) [52,53]. 

A diet enriched in PUFAs or SFAs has a distinct effect on liver metabolism and visceral fat accumulation because SFAs result in a significant increase in liver and visceral fat compared to PUFAs. Preclinical studies illustrated that a diet rich in n-3 PUFAs, like docosahexaenoic acid (DHA), suppresses diet-induced steatosis, inflammation, and fibrosis [54], along with a positive effect on insulin resistance and serum triglycerides [55,56]. Furthermore, a preclinical study highlighted that, among 3-PUFAs, DHA is superior to eicosapentaenoic acid (EPA) to reduce NASH related inflammation and fibrosis, as well as hepatic fat content [57,58]. 

The harmful role of SFAs compared to PUFAs has also been confirmed by Bjermo et al. who analyzed two hypercaloric diets, one enriched in n-6 PUFA and the other in SFAs, and showed that, after seven weeks, a similar weight gain was observed, with a dramatically increment in hepatic fat and a two-fold increase in visceral fat only in patients assigned to the SFAs diet compared to the PUFAs diet [59]. In addition, a 10-week randomized clinical trial comparing two isocaloric diets containing n-6 PUFA or SFAs showed an improvement in metabolic status and a decrease in hepatic fat using n-6 PUFAs diet, despite no weight modification [60]. 

Besides PUFAs, a protective role in fat accumulation has been demonstrated also for MUFAs. In fact, an energy balanced diet with a high proportion of MUFAs was associated with a reduction in hepatic fat in patients with prediabetes [61], type 2 diabetes [62], and NAFLD [63], differently from an energy balanced diet with a very high proportion of SFAs. Indeed, the EASL–EASD–EASO Clinical Practice Guidelines recommended the Mediterranean diet for NAFLD patients in consideration of its high content of MUFAs, present in olive oil and nuts, and the large amount of fruits, legumes, vegetables, and fish and low in red and processed meats and sweets [64]. 

Multiple checkpoints are influenced by the high-fat diet and oxidative stress is directly associated with an excess of n-6 PUFAs and a low amount of n-3 PUFAs, generating lipid oxidation and NAFLD progression [65]. Firstly, diet modulates gut microbiota, whose metabolic products are delivered to the liver by the portal vein. The alterations of intestinal microbiota, characterized by overgrowth of some bacteria phyla and alteration of epithelial barrier permeability, contribute to inflammation, insulin resistance, and fat accumulation. Consequently, these modifications lead to obesity and NAFLD, dysregulating liver functions [66]. Secondly, SFAs promote mitochondrial dysfunction, altering respiratory chain activity and ATP homeostasis, increasing formation of reactive oxygen species (ROS) and inducing apoptosis, consequently impairing phospholipid metabolism and increasing insulin resistance [67]. Lastly, high-fat diet enriched with SFAs was hypothesized to trigger de novo lipogenesis through activation of the peroxisome proliferator-activated receptor (PPARγ)-coactivator-1beta (PGC-1) and the enhancement of transcription in genes involved in lipid metabolism, like the stearoyl-CoA desaturase-1, fatty acid synthase, and diacylglycerol acyltransferase compared to a PUFA diet [68]. On the contrary, n-3 PUFAs control the activity of transcription factors regulating the expression of gene encoding for de novo lipogenesis, fatty acid oxidation, fat uptake from the circulation and VLDL assembly and secretion [54]. Moreover, n-3 PUFAs have also been hypothesized to attenuate fibrosis, possibly by reducing the expression of a broad array of proteins involved in fibrosis [58]. Moreover, DHA has been supposed to act directly on human stellate cells suppressing the expression of Col1A1, the major collagen subtype induced in rodent and human NASH [69]. However, future studies are needed to clarify the role of DHA in hepatic fibrosis.

### 3.2. Lipids and Cardiovascular Disease

Cardiovascular disease (CVD) is the first cause of death and disability worldwide, and diet is one of the most important modifiable risk factors [70]. 

As described in the previous paragraph, in the last few decades, the daily diet is dramatically changed increasing the proportion of fatty acids, which nowadays represent 28–42% of total energy consumed by European populations [71], whereas in the past was approximately 20–30% of total energy [72]. Moreover, the kind of fatty acids in the diet is mutated, n-3 PUFAs intake is reduced, and the ratio between n-6 and n-3, which was 1:1, nowadays is dramatically increased to a range between 10:1 and 20:1 [72]. Several studies illustrate that a low ratio between n-6 and n-3 PUFAs has been considered protective for the development of many diseases, such as cardiovascular disease, cancer, and metabolic disease [73]. The cardioprotective action of the eicosanoids derived from n-3 PUFAs is justified by their anti-inflammatory, antiatherogenic, antiarrhythmic, antithrombotic/antiplatelet, antioxidant, hypotensive effects, and the positive action on lipid metabolism [74]. 

Regarding the food rich in n-3 PUFAs, the consumption of tree nuts, for example, almonds, reduces LDL cholesterol levels by 3 to 19% [75], while fish consumption has beneficial effects on insulin sensitivity, type 2 diabetes mellitus (T2DM), and lipid profile [76]. In addition, 3-PUFA contained in fish have been associated with a reduced risk for death from coronary heart diseases in healthy individuals [74,77], and in particular the α-linolenic acid with a reduced risk of myocardial infarction [78]. 

According to the literature consensus, healthy adults should have a daily intake of at least 0.5–1 g/d n-3PUFAs, equivalent to 2–4 servings per week of fish or a half serving of oily fish, in order to reduce the risk of CVD and improve the features of metabolic syndrome [79]. Indeed, conversely to the past when the total intake of n-3PUFAs exceeded 5–6 g/d [80], nowadays only the traditional Japanese diet contains that level [81]. 

Moreover, many epidemiological studies demonstrated a low cardiovascular risk associated with Mediterranean diet. In particular, the most important factor is the daily consumption of olive oil, the greatest source of dietary MUFA (about 90% of all MUFA). In fact, the PREDIMED trial (Primary Prevention of Cardiovascular Disease with a Mediterranean Diet) demonstrated that extra-virgin olive oil or nuts in supplementation to Mediterranean diet reduced the incidence of major cardiovascular events [9]. 

The protective role of an adequate consumption of olive oil (23 g/day) in the CV risk has already been recognized at the end of the last century by the Food and Drug Administration, addressing olive oil as a qualified dressing able to decrease the risk of coronary heart disease [82]. Indeed, olive oil has several beneficial effects on the cardiovascular system: decreases oxidative stress, inhibits oxidation of LDL-cholesterol, cuts-down serum level of triglycerides [83], modulates inflammation [84], reduces blood pressure, delays gastric emptying, decreasing postprandial hyperglycaemia, and improves glycaemic control in diabetic patients [85]. 

If on one hand MUFAs reduce CV risk, on the other hand, SFAs are associated with an unfavorable metabolic and CV profile, especially because of the increase in low density lipoprotein (LDL) cholesterol. In consideration of that, International Guidelines strongly recommended to reduce the daily consumption of SFAs to a maximum of 5–6% of the total lipid intake [86]. 

## 4. Role of Proteins in NAFLD and Cardiovascular Disease

### 4.1. Proteins and NAFLD

Data on the effects of dietary protein in patients with NAFLD are limited and controversial, while, in animal models, results suggest that dietary proteins have a beneficial effect on glucose metabolism, are essential for the regeneration of hepatocytes and could counteract the development of steatosis providing the amino acids needed for the inclusion of fat in lipoproteins and favoring its export from the liver. In addition, the energy requirement for amino acid catabolism could lead to oxidation of hepatic lipids with a consequent increase in fat depletion from the liver [87,88]. On the contrary, in population studies, diets enriched in animal proteins, especially in overweight patients, are associated with an increased risk of NAFLD development [89]. However, in the few available controlled trials, diets enriched in proteins were often also low-caloric diets, thus it is not possible to draw any firm conclusions.

Recent studies have highlighted sarcopenia as a risk factor for disease severity in NAFLD. In sarcopenic obesity, defined by the concomitant presence of sarcopenia and obesity, the coexistence of muscle loss and fat accumulation accelerates insulin resistance and inflammation, which lead to atherosclerotic damage and a more severe liver disease (NASH and advanced fibrosis) [90,91].

Very recently, nutritional and behavioral models have been proposed to contrast the increased risk of frailty in the obese, diabetic, and elderly patients (>75 years old) in whom quantities and qualities of various nutrients, including proteins, have been modified compared to younger subjects. These categories of patients should undergo anthropometric measurements and muscle function tests to allow targeted early nutrition interventions (i.e., 1.5 g/kg/day of protein intake, 2–3 hourly food intake with kidney function monitoring) and promotion of physical exercise [92].

### 4.2. Proteins and Cardiovascular Disease

Several large cohort observational and intervention studies in both Western and Asian populations have evaluated whether plant and animal proteins differ in the reduction/promotion of CV risk [93,94]. However, evidence is not definitive because it is difficult to isolate the independent effects of specific proteins from the contribution of other non-protein components in both plant and animal food source, the content of particular amino acids and the interaction with the intestinal microbiome. Red meat, for example, is rich in proteins but also in cholesterol and heme-iron, nutrients associated with the development of CVD through different mechanisms (increase of free fatty acids, oxidative stress, free radical production) [95,96]. In addition, as reported above, sarcopenia also, consequent to protein deficiency, is a risk factor for atherosclerosis, thus increasing CV risk [90]. 

## 5. Role of Fibers in the Setting of NAFLD and Cardiovascular Disease

### 5.1. Fibers and NAFLD

The role of fibers in NAFLD has been studied in animal models and in patients. However, results do not allow for understanding whether the impact of fibers is a direct consequence of the modulation of gut microbiome or if fibers, by themselves, reduce inflammation and thus counteract the development of NASH. Fibers can be distinguished into soluble and insoluble, and both can be non-fermentable and fermentable.

Cellulose, a type of insoluble non-fermentable fiber, exhibits protective anti-inflammatory effects through alteration of microbiota [97]. In mice, low-cellulose diets alter the intestinal mucosa by inducing crypt atrophy and reduction of goblet cells, resulting in development of inflammation. Conversely, diets rich in cellulose reduce IL-1β and TNF-α, both mediators of inflammation. Furthermore, it has been shown in mouse models of endotoxemia that a diet rich in cellulose acts by reducing the macrophage activity of the spleen and by attenuating the activity of hepatic NF-κB [98]. In addition, most soluble fibers are fermented by gut microbiota to produce metabolites that impact metabolic processes.

For example, inulin is a type of soluble fiber, the activity of which depends on its chemical structure and the presence of inulin in the diet of overweight, non-diabetic patients, induces a reduction in insulin resistance, coinciding with a change in the gut bacterial population [99]. In addition, β-glucan supplementation, derived from plant or fungal cell walls, and considered a soluble fiber, reduces insulin resistance, dyslipidemia, and hepatic steatosis jointly with alterations of gut microbiome [100]. 

### 5.2. Fiber and Cardiovascular Disease

The benefic effect of fibers is documented by the evidence that vegetarian diets reduce atherosclerotic cardiovascular damage with a significant decrease in coronary artery calcifications [101]. In a meta-analysis of nine cohort studies including 222,081 men and women, the overall reduction in coronary heart disease risk was 7% for each additional portion of vegetable intake per day [102]. Dark green leafy vegetables had the highest reduction in coronary heart disease risk, but it has to be stressed that vegetarian diets are often deficient in many nutrients that have to be supplemented.

## 6. Role of Micronutrients in NAFLD and Cardiovascular Disease

The potential role of micronutrients in both NAFLD and CVD has been debated for years and, although hundreds of papers have been published, sound results are still not available as proven by the fact that very rarely do these compounds reach clinical practice. 

The main results reported in literature on bioactive compounds will be described below. The best results on the micronutrients utilization derive from cellular or animal models, while those obtained in patients often are conflicting. In many studies, multiple compounds are combined to enhance the antioxidant, anti-inflammatory, and anti-fibrogenic properties of individual compounds making even more difficult to understand the effect of the individual compounds.

Vitamin E is a fat-soluble vitamin that works as an antioxidant. It was evaluated alone or in combination with other antioxidants such as Sylimarin. The steatohepatitis study (PIVENS) showed that supplementation of 800 IU vitamin E (α-tocopherol) per day (in people without diabetes) improved steatosis and inflammation and induced resolution of NASH [103]. However, a long-term risk of increased mortality has been demonstrated at these dosages. A recent meta-analysis of 16 controlled studies showed that low doses of vitamin E alone would reduce the risk of myocardial infarction (RR 0.82; 95% CI, 0.70–0.96; *p* = 0.01) [104]. Furthermore, supplementation with Vitamin E is associated with a 2.5% increase in flow-mediated vasodilation and consequently a reduction in CV risk [105].

Silymarin, one of the most studied nutraceutical of plant origin, has been shown in numerous studies to have several positive effects on the liver and on the cardiovascular system: anti-oxidant (direct scavenger activity, optimization of mitochondrial function), anti-inflammatory (inhibition of NF-kB activity, reduction of the synthesis of proinflammatory cytokines IL-1, IL6, TNFa, TNFb), anti-apoptotic activity (modulation of caspase release) antifibrotic (inhibition of the conversion of stellate cells to fibroblasts, downregulation of target genes) and choleretic (upregulation of the bile salts export pump). Finally, it also exerts a favorable metabolic action by fostering the PPAR-agonist activation and increasing the expression of Glucose transporter-4 (GLUT4) on the cell surface, inhibition of Hydroxyl-Methyl-Glutaryl-Coenzyme-A reductase).

The studies that have documented the effect of silymarin (or its isoforms) in vivo have used this compound in monotherapy or in association with Vitamin E, and have demonstrated normalization of transaminases, reduction of GGT, and of the degree of steatosis (evaluated by ultrasound), improvement of fasting blood glucose and insulin resistance. However, these positive effects disappear upon discontinuation of the drug [106].

The combined use of vitamin E and Sylibin, the main active compound of sylimarin, in addition to a low-calorie diet is associated with a reduction of transaminases (ALT and AST), GGT and hepatic steatosis index, while results on HOMA, BMI, and lipids are conflicting. A mixture of vitamin E and C (600 IU/day and 500 mg/day, respectively) given to patients with elevated levels of ALT and biopsy-proven NAFLD that followed a weight-reducing diet showed a significative reduction of transaminases and of fibrosis [107]. It has also been reported that addition of atorvastatin to vitamin C and E would reduce the risk of fatty liver development [108].

## 7. Vitamin D 

Vitamin D is a fat soluble secosteroid hormone, about 10% derived from the diet, while 90% from the cutaneous conversion of 7-dehydrocholesterol to cholecalciferol due to exposure to ultraviolet B rays. 

Animal and cellular studies showed that vitamin D plays a fundamental role in the metabolism of minerals, but also in inflammation, regulation of the immune response and cell differentiation, with impact on the liver and on the cardiovascular system [109].

An interaction between vitamin D and NAFLD has been described. Recently, some literature data showed a higher prevalence of vitamin D deficiency in NAFLD, with vitamin D playing a role in NAFLD and cardiovascular disease through increasing insulin sensitivity, reducing inflammation of the adipose tissue, reducing liver inflammation and fibrosis [110]. However, it is still debated whether patients with NAFLD have a higher prevalence of vitamin D deficiency than people without steatosis and the effectiveness of adding vitamin D on NAFLD histological damage [111]. 

The decrease in sun exposure and the modern lifestyle, besides the reduction of the hepatic and renal hydroxylation capacity with aging, causes vitamin D deficiency in a large part of the population. Vitamin D deficiency has been reported in chronic liver diseases of different etiology, and it has even been associated with hepatic decompensation in liver cirrhosis. However, the most reliable data demonstrate effectiveness of vitamin D supplementation when associated with diet, thus it is not clear which is the real impact of vitamin D by itself.

## 8. Vitamin K

The role of vitamin K on CV alterations is documented in cohorts of patients with CVD, hypertension, and in the general population. The Multi-Ethnic Study of Atherosclerosis (MESA) demonstrated an increased prevalence of CAC in patients with a low intake of vitamin K1 with diet, especially in patients on antihypertensive drugs. A population-based study of 4807 participants found that the incident risk for chronic heart disease was reduced by Vitamin K [112,113].

## 9. Curcumin

Curcumin is an insulin sensitizing agent extracted from *Curcuma longa*, whose effect on NAFLD is reported in few preclinical and clinical studies. Curcumin would reduce some altered parameters of NAFLD including transaminases, abdominal circumference, and especially ultrasound steatosis. However, given the high dosage needed to obtain the effect, the only recent availability of pharmaceutical preparations with a known dose of the drug and finally the presence of a few randomized controlled trials with adequate numbers of patients, make these data still preliminary [114,115]. 

Curcumin displays a role also in CVD. In fact, in mice models, it significantly attenuates collagen deposition and inhibits cardiac fibroblast proliferation and migration after coronary damage [116]. In a study of 121 patients, curcumin reduced myocardial infarction after coronary artery bypass graft from 30% to 13% [117]. 

The potential effect on NAFLD of the administration of the mixed extracts obtained from natural sources was also evaluated. In general, these mixtures are very rich in phenolic compounds, carotenoids, vitamins, and minerals. Among them, some preparations were used more rigorously. Most of the beneficial effects of curcumin or its bioactive agents are still limited to preclinical studies in animal models [118], while randomized, controlled studies in humans have not reported conclusive results even for the small sample size [119].

## 10. Polyphenols

Polyphenols derive from plant metabolism and are divided into flavonoids and non-flavonoids and are contained in many natural compounds, as vegetables, cereals and fruits), as well as in tea, coffee, red wine, and beer. Normally, the daily diet intake is around 800–1300 mg [120] and their supplementation is essential because of their antioxidant, anti-inflammatory, anti-mutagenic and immunomodulatory action. As a consequence, a diet rich in polyphenols would reduce dyslipidemia and oxidative stress [121]. There are numerous studies in animal models: polyphenols can decrease the transcription of SREBP-1c and determine an increase in the transcription of PPAR-a, reducing insulin resistance and inflammation. However, clinical data on patients are conflicting. Some clinical studies which evaluated the effect of anthocyanins, phenolic acids, and catechins in NAFLD, found a reduction in biomarkers of hepatocyte apoptosis, ALT levels and liver fat content, evaluated by CT [122]. Resveratrol is currently one of the most evaluated polyphenols and some studies have been conducted in NAFLD with opposite results, showing a transient reduction in ALT and AST and CK18, but not of fibrosis [123]. 

It is therefore possible to hypothesize, on the basis of the data reported in the literature, that catechins and anthocyanins could have some effect on steatosis; however, data need to be confirmed in further research.

## 11. Caffeine

Several studies have evaluated the efficacy of caffeine, an alkaloid derivative of xanthine, which is the main compound in coffee, and of coffee consumption itself, in NAFLD. A reduction in the risk of developing NAFLD of approximately 30% has been described. In addition, a reduction in fibrosis has also been reported, the mechanism of which could be linked to inhibition of adhesion and activation of hepatic stellate cells. However, the definition of regular coffee consumption varies widely between studies and this may represent a limitation of the results presented in different meta-analyses [124,125]. It is also unclear whether the beneficial effect of caffeine/coffee is due to an association rather than a real causal mechanism. Finally, it is possible that other compounds present in coffee, including polyphenols and melanoidins, are the real culprits.

## 12. Genetics and Diet in NAFLD and Cardiovascular Disease

As already said, NAFLD is characterized by metabolic alterations, an incorrect diet, mainly rich in SFA and sugars, and physical inactivity. In addition, patients with NAFLD and metabolic alterations are exposed to high cardiovascular risk and a high rate of CV events. However, not all patients with a risky metabolic profile or unhealthy lifestyle develop hepatic steatosis or a cardiovascular disease. 

### 12.1. Genetic Bases of NAFLD and CV Risk

The role of genetic predisposition is well established in the pathogenesis of NAFLD, and the strong heritability component is sustained by epidemiological, familial, and twin studies [126]. Several single nucleotide polymorphisms (SNPs) in genes involved in lipid metabolism play a role in the onset of NAFLD and progression towards advanced forms of liver damage. The patatin-like phospholipase domain-containing 3 (PNPLA3) is the major determinant of hepatic fat content and of its progression [127,128] and leads to accumulation of a mutated protein, with an impaired function in lipase activity on hepatocellular lipid droplets, thus favoring triglycerides accumulation within the liver. Another genetic determinant in NAFLD is the variant in a transmembrane 6 superfamily member 2 gene (TM6SF2) which predisposes to an increased hepatic fat content because of retention of lipids and impairment in very low-density lipoprotein (VLDL) release by the liver [129]. In addition, mutations in the membrane bound O-acyltransferase domain-containing 7 gene (MBOAT7) foster liver fat accumulation and inflammation. In fact, this gene encodes for an enzyme localized on the mitochondria-associated membrane, where lipid droplets formation occurs, thus regulating the desaturation of membranes phospholipids and the production of arachidonic acid, which is involved in inflammatory processes [130]. Finally, the glucokinase regulatory protein (GCKR) gene, which modulates the glucose influx into the hepatocytes and the de novo lipogenesis, is involved in NAFLD development [131]. However, less than 10% of inherited variability is explained by these common variants.

**Figure 1:**
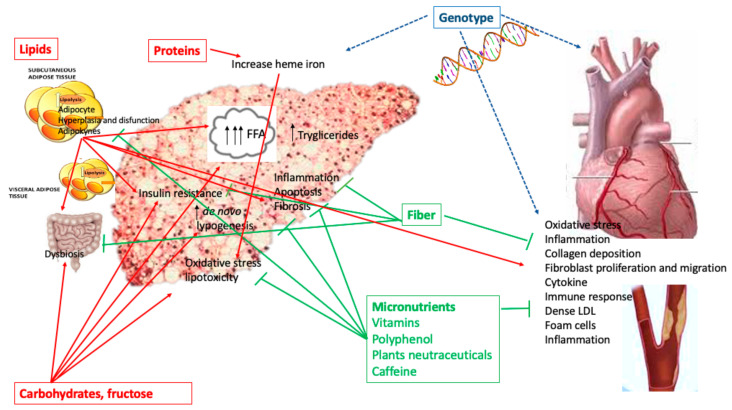
Potential multiple distinct and interrelated mechanisms by which nutrients promote (red line) or prevent (green line) liver and cardiovascular damage. Influence of genetics on NAFLD and cardiovascular damage (blue line).

Likewise, a large number of genes and their variants may influence the CV risk by different routes. Among them, the 5–10-Methylene tetrahydrofolate reductase (MTHFR) and the Methionine synthase (MTR), both regulating homocysteine metabolism, an atherogenic and prothrombotic protein, have been related with an increased risk of coronary artery disease (CAD) [132,133]. Similarly, the LDL receptors gene which is involved in low-density lipoprotein (LDL) and triglyceride metabolism and the LIPC gene (hepatic lipase C), the major determinant of plasma HDL concentration, are linked to increased prevalence of myocardial infarction and CAD in the general population [134,135]. Finally, the apolipoprotein APO-A and APO-E family gene, encoding key regulators of plasma lipids, have a significant impact on CAD [136]. The possible mechanisms and interactions between liver, cardiovascular, and nutrient damage are shown in Figure 1.

### 12.2. Nutritional Genomics or Nutrigenomics

Besides pure genetic bases of diseases, in the last few years, evidence is accumulating on the complex interplay between genetic predisposition and environmental influences, particularly diet, in the development of NAFLD as well as in the increase in cardiovascular risk, possibly suggesting common etiopathogenetic pathways linking both hepatic and CVD. 

Therefore, since 2013, the term nutritional genomics or “nutrigenomics” has been coined, defining the interaction between cellular, genetic processes, and nutritional environment [137]. Nutrigenomic has the aim of exploring the effect of diet on disease development by modulating the expression of an individual’s genetic pattern. 

Three main fields of study cover this interaction: (1) the genetic variability response towards nutrition, named nutrigenetics; (2) the effect of nutrients on DNA metabolism; and (3) the effect of nutrients on genetic expression.

#### 12.2.1. Nutrigenetic: Genetic Variability Response towards Nutrition

The most widely studied gene which interacts with the environment in NAFLD is represented by PNPLA3. In fact, accumulation of the protein encoded by the mutated gene is triggered by acquired factors, like obesity [138], alcohol consumption [139], and diet, especially with a high content in carbohydrates [140]. A nutrigenetic analysis in a cohort of Italian adolescents showed that hepatic triglycerides accumulation was related to carbohydrate and sugar intake in the homozygous mutated group but not in the wild type group [141]. The rationale of this influence relies on carbohydrate-mediated upregulation of PNPLA3, which in turn favors the accumulation of the pathological protein on the surface of lipid droplets. 

Indeed, PNPLA3 is influenced also by fatty acids in the diet, particularly n-6/n-3 PUFA [142]. In 127 Caucasian adolescents with steatosis evaluated by MRI, increased transaminases and free fatty acids were associated with a diet with a high n-6/n-3 PUFA ratio, but only in patients homozygous for the mutated PNPLA3 variable. This result is possibly related to the impaired hydrolyzing function of PNPLA3, with accumulation of hepatic triglycerides and metabolites of omega 6 PUFA with pro-inflammatory action (Santoro, 2012) [142]. In addition, patients with NAFLD and mutated PNPLA3 seem to have no response in terms of reduction of the novo lipogenesis and improvement in transaminases by n-3 PUFA supplementation in the diet [143]. 

In addition, mutated patients for the TM6SF2 and MBOAT7 genes are affected by diet. Carriers of the mutated TM6SF2 allele present better fasting and postprandial lipid profiles, with consequent reduction of circulating atherogenic lipoproteins even after a high fat challenge compared to the wild types [144,145]. As regarding MBOAT7, besides the mutations associated with genetic predisposition, its expression may be downregulated also by diet-induced hyperinsulinemia [146], thus fostering the accumulation of phosphatidylinositol species which are used as substrates for the synthesis and hepatic deposition of saturated and mono-unsaturated triglycerides. Finally, several studies have shown that mutated allele carriers of GCKR present lower fasting glucose and insulin concentrations after a whole-grain supplementation independently of other metabolic risk factors, as well as to dietary restrictions [147]. 

In the setting of CVD, the MTHFR gene suffers from folic acid deficiency, being predisposed to systemic inflammation and prothrombotic events [132]. Another gene named Transcription factor 7-like-2 (TCF7L2) and implicated in type 2 diabetes mellitus interacts with the Mediterranean diet in the reduction of stroke risk [148]. 

The benefit of PUFAs in CVD is widely known [149]. The effect of PUFA intake on HDL cholesterol concentration is mainly modulated by the APOA1 (main apolipoprotein of HDL), thus it was shown that PUFAs consumption was associated with higher HDL cholesterol concentration depending on APOA1 polymorphism [150]. In addition, the APO-E4 polymorphism has been associated with the development of CAD. A study examining the effect of a quercetin supplementation (flavonoid glycosides) in a cohort of overweight-obese individuals with metabolic syndrome showed a significant reduction in HDL in the subgroup of patients carrying the APO-E4 polymorphism, differently from the APO-E3 carriers [151]. In another population-based study involving nearly 2000 subjects, the APO-E3 and APO-E4 genotypes were significantly associated with a high risk of acute myocardial infarction in patients treated with high saturated fatty acids (>10%) diet compared with APO-E2 carriers [152]. 

#### 12.2.2. Effect of Nutrients on DNA Metabolism

Studies of the effect of micronutrients on DNA metabolism have shown that several micronutrients (especially calcium, folate, nicotinic acid, vitamin E, retinol) are necessary for DNA homeostasis, being cofactors of a several enzymes essential to DNA synthesis and repair [153], whereas others, as the TFAs, favor DNA damage [154]. Their depletion may prompt unfavorable DNA modifications. 

In addition, telomeres shortening is typical of aging and of many chronic diseases, including NAFLD [155] and cardiovascular disease [156]. Diet has an impact on telomeres homeostasis, with vegetables, fibers, and omega-3 fatty acids preventing telomeres shortening, whereas saturated fatty acids, processed meat, or carbohydrates with a high glycemic index favoring it [157,158]. 

#### 12.2.3. Effect of Nutrients on Genetic Expression

The study of the effect of nutrients on gene expression primary identifies the transcription factors that are sensitive to a specific nutrient and their target genes, then the signaling pathways activated by these transcription factors and the possible proteins and metabolites created, detected by “omics” techniques, and finally the organ-specific response to nutrients [159]. 

Many transcription factors are susceptible to diet influences. In particular, the peroxisome proliferator-activated receptor (PPAR) family, which is involved in glucose and lipid metabolism, adipocyte differentiation, and inflammatory response, is one of the most widely studied. In fact, each PPAR family member α, δ, and γ is responsive to specific dietary fatty acids (PUFAs, monounsaturated fatty acids, and SFAs) [160,161]. Interestingly, PPARγ expression induced by omega 3 PUFA can increase the lipid storage capacity of adipose tissue along with hepatic fatty acid oxidation and reduce systemic inflammation, with consequent improvement in insulin sensitivity, hepatic steatosis, and CV risk. Polymorphisms in PPAR-γ2, the most important form associated with CVD, have been analyzed in a 2-year nutritional intervention with a Mediterranean-style diet vs. conventional low-fat diet in 774 patients at high-cardiovascular risk [162]. The results showed that only carriers of the mutated allele allocated in the low-fat diet and not in the Mediterranean diet arm significantly increased waist circumference compared with non-mutated participants. Therefore, it could be speculated that the Mediterranean diet could mitigate the adverse metabolic effects of mutation in PPAR-g2 gene in a high-cardiovascular risk population. 

### 12.3. Epigenetic, Diet, NAFLD, and CVD

Along with genetic predisposition to NAFLD and CVD, disease development and natural history are also modulated by epigenetic mechanisms, heritable but reversible phenomena that modify gene transcription without altering DNA sequence [163]. 

The epigenetic modulation of gene expression includes (a) DNA methylation; (b) chromatin remodeling (histone modifications); and (c) microRNAs formation (small particles of non-coding RNA with specific functionality). Epigenetic modifications affect hepatic lipid metabolism, insulin resistance, mitochondrial function, and oxidative stress and diet may modulate all these [164,165]. 

(a) Some nutrients, such as choline, methionine, folate and vitamin B12 are considered “methyl-donor” promoting methylation of DNA and histones. Actually, DNA methylation, considered a key factor in the progression from simple steatosis to NASH and in triglycerides metabolism, may be increased by dietary deficiency of choline, betaine, B12, and folate. In fact, their supplementation is associated with an increase in the hepatic efflux of triglycerides [166]; conversely, their deficiency promotes hepatic triglycerides accumulation by fostering over-expression of genes involved in fatty acids synthesis [167]. In addition, in a rodent model, supplementation of methyl-donor micronutrients to a high-fat-sucrose diet mitigated the effect on triglycerides accumulation within the liver through a hypermethylation of a key enzyme involved in fatty acids synthesis [168]. In vivo studies showed the epigenetic effect of these nutrients, altering endothelial cell function, and inflammation processing, with consequent plaque formation and increased CV risk [169].

(b) Post-translational modification of histones is essential to define chromatin architecture and thus gene accessibility. In response to caloric restriction, Sirt1, a key enzyme involved in DNA repair, induces histone and non-histone deacetylation of genes involved in insulin-resistance and inflammation of the adipose tissue, promoting gluconeogenesis and fatty acid oxidation in the liver [170]. A histone acetyltransferase inhibitor, the tannic acid, a plant-derived polyphenol, has been demonstrated to block the hyperacetylation of histones of the promoter of DNL, thus reducing its activity and hepatic fat accumulation [171]. Likewise, SFAs, produced by fermentation of polysaccharide by gut microbiota, also inhibit histone deacetylases with consequent impairment in metabolic and inflammatory processes [172]. 

(c) Alteration of mRNA stability may arise through formation of small RNA molecules called microRNAs which bind to transcriptional mRNA turning into post-transcriptional repression of targeted protein-coding genes. Unhealthy diets may foster NAFLD development by altering miRNAs. One of the most represented in liver and hallmark of fat accumulation and steatosis severity, the miR-122 has been shown to be responsive to diet either in vitro or in vivo models and in humans. In particular, a high-fat-diet upregulated miR-122 with consequent promotion of lipogenesis within the liver [173]. However, non-conclusive data were reported on plant-derived dietary microRNAs which may potentially cross the intestinal and once in the bloodstream regulate host gene expression involved in cardiometabolic risk [174]. On the other hand, host derived small RNAs regulating processes of relevance to cardiometabolic risk are altered by dietary intervention [175]. 

Interestingly, epigenetic mechanisms may arise during the gestational period, as the fetus liver may be “primed” with unhealthy metabolic pattern by mother over-nutrition, as well as in post-natal period and over time with aging. For example, in pregnant women with a diet rich in fat, a state of chronic-low grade inflammatory arises in the placenta as well in the fetal organs, including the liver, contributing to steatosis development [176]. 

Based on this background, the role of gene–diet interaction in patients with NAFLD is crucial because, by modifying dietary pattern in genetically predisposed individuals, it could be possible to modulate specific clinical outcome. The long-term hypothetical objective could be the tailored therapy based also on a personalized nutritional approach.

## 13. Preliminary Data

In order to evaluate the role of diet on clinical presentation and cardiovascular alterations in patients with NAFLD, we enrolled 154 newly diagnosed, consecutive, untreated NAFLD patients (M/F 112/42 mean age 49 ± 12 years), who were administered a semi-quantitative food-frequency questionnaire (dietary record including 118 food items covering seven days) to calculate energy and nutrients intake. Steatosis, carotid intima-media thickness (IMT), and plaques were evaluated by ultrasonography; diastolic dysfunction (E/A < 1), left ventricular mass, and epicardial adipose thickness (EAT) by echocardiography. Twenty-three (15%) patients had “lean” NAFLD (BMI < 25 kg/m^2^). Moderate to severe steatosis was present in 61%, IMT above normal value in 13%, carotid plaques in 28%, and E/A < 1 in 39%; mean EAT was 5.8 ± 4 mm. Total calories and macro-nutrient intake (g/day) were: 1705 ± 5 750 kcal; proteins 98 ± 46, fat 65 ± 32 (saturated 23 ± 14; PUFA 10 ± 5; monounsaturated 25 ± 12), carbohydrates 164 ± 75 (simple sugar 64 ± 35), fibers 23 ± 14. Interestingly, no difference in daily dietary composition of nutrients and total Kcal between lean and overweight patients was observed, despite a higher prevalence of metabolic comorbidities and moderate-severe steatosis in the latter. Among nutrients, fructose intake was significantly higher in patients with plaques (*p* = 0.03) and vitamin E lower in those with E/A < 1 (*p* = 0.01). At multivariate analysis adjusted for age, sex, and BMI, diet fat content was significantly associated with dyslipidemia (OR 1.2, 95%CI 1–1.4, *p* = 0.04), fat, and carbohydrate with moderate-severe US steatosis (OR 1.2, 95%CI 1.03–1.5, *p* = 0.01). 

## 14. Conclusions

In conclusion, both diet and genetic factors strongly impact on both NAFLD and cardiovascular damage, either alone or when interacting. 

In particular, solid data in literature highly stress the potentially negative role of an incorrect diet, rich in fructose and added sugars, as well as in n-6 PUFAs and SFAs, in the development of both hepatic and cardiovascular diseases. Therefore, the elimination of soft drinks and sweetened bakery products, along with dairy and animal fat, from daily consumption, preferring complex carbohydrates, possibly whole grain, fibers, olives, and vegetable oils, fish, seeds, and nuts rich in MUFAs, and n-3 PUFAs is highly advisable. Indeed, one of the most recommended diet for patients with NAFLD is the Mediterranean diet, which has also been demonstrated to dramatically reduce cardiovascular events. If the role of carbohydrates and fat is well established, that of proteins and micronutrients (especially vitamins) is not completely elucidated, despite a protective role mediated by antioxidant and anti-inflammatory actions having been suggested. 

Along with the diet, some genetic bases, either congenital or caused by environmental actions (i.e., epigenetic), have been implicated in the pathogenesis and progression of hepatic and cardiovascular disease. Even more interestingly, in the last few years, evidence is accumulating on the interplay between genetic predisposition and environmental influences, particularly diet, in the setting of NAFLD and cardiovascular disease, coining the new concept of nutritional genomics or nutrigenomics. 

Based on this evidence, an accurate correction of patients’ diet along with the understanding of the gene-diet interaction become crucial because modification of dietary pattern in genetically predisposed individuals could modulate specific clinical outcomes, either the metabolic, hepatological, or cardiovascular ones, so that a personalized nutritional therapy should be speculated on in the future. 

## Abbreviations

NAFLD	non-alcoholic fatty liver disease
NASH	non-alcoholic steatohepatitis
CVD	cardiovascular disease
FFAs	free fatty acids
TFA	trans fatty acids
PNPLA3	patatin-like phospholipase domain containing 3
TM6SF2	transmembrane 6 superfamily member 2
MBOAT7	membrane bound O-acyltransferase domain-containing 7
HFCS	high fructose corn syrup
DNL	de novo lipogenesis
CAD	coronary artery disease
APO	apolipoprotein
SFAs	saturated fatty acids
MUFA	monounsaturated fatty acids
PUFA	unsaturated fatty acids
MTHFR	methylene tetrahydrofolate reductase
DHA	docosahexaenoic acid
EPA	eicosapentaenoic acid
PPAR	peroxisome proliferator activated receptor
HFCS	high fructose corn syrup
GLUT4	glucose transporter-4
